# Laboratory Markers Associated with Deep Neck Infection in Emergency Department Patients with Throat or Neck Symptoms

**DOI:** 10.3390/diagnostics16183016

**Published:** 2026-09-17

**Authors:** Jeong-Hwan Shin, Tae-Youn Kim, Je Seop Lee

**Affiliations:** 1 Department of Emergency Medicine, Inha University Hospital, Incheon 22332, Republic of Korea; docsjh87@gmail.com; 2 Department of Emergency Medicine, Inha University Hospital, Inha University College of Medicine, Incheon 22212, Republic of Korea; suffo41@inha.ac.kr

**Keywords:** deep neck infection, emergency department, computed tomography, absolute neutrophil count, C-reactive protein, erythrocyte sedimentation rate, inflammatory markers

## Abstract

**Background/Objectives**: Deep neck infection (DNI) can be difficult to distinguish from localized upper aerodigestive disease during emergency department (ED) evaluation. We assessed routinely available laboratory markers in adults presenting with throat or neck symptoms and examined whether the associations were reproduced in an imaging-defined subgroup. **Methods**: This single-center retrospective cohort included 1640 adults with prespecified throat- or neck-related presentations between January 2021 and May 2026. The primary outcome was DNI confirmed using prespecified diagnostic evidence from contrast-enhanced neck CT, endoscopic/fibroscopic evaluation, or ultrasonographic evaluation with aspiration or drainage when applicable. A clinical model incorporating demographics, presenting symptoms, symptom duration, and initial vital signs was compared with the same model after addition of inflammatory laboratory markers. A secondary analysis was restricted to 662 patients who underwent contrast-enhanced neck CT. **Results**: DNI was confirmed in 273 patients (16.6%). In 1166 complete cases (268 events), the 10-fold cross-validated AUC increased from 0.644 for the clinical model to 0.788 for the clinical model augmented with ANC and CRP (ΔAUC, 0.144; 95% CI, 0.109–0.179; likelihood-ratio *p* < 0.001). In this augmented model, ANC (aOR per 5 × 10^9^/L, 1.49; 95% CI, 1.25–1.79) and CRP (aOR per two-fold increase, 1.51; 95% CI, 1.37–1.65) remained associated with DNI. In the CT-defined subgroup, the corresponding cross-validated AUC increased from 0.562 to 0.685 (ΔAUC, 0.123; 95% CI, 0.077–0.167). Derived inflammatory indices showed no clear advantage over conventional markers. **Conclusions**: ANC and CRP remained associated with confirmed DNI after adjustment for clinical characteristics, and their addition improved discrimination beyond structured clinical assessment. More complex derived indices offered no clear advantage over conventional markers. Model performance remained insufficient for stand-alone diagnostic or imaging decisions.

## 1. Introduction

Throat and neck symptoms are common presentations in emergency department (ED) practice [1], and their differential diagnosis ranges from localized upper aerodigestive tract infections to deep neck infection (DNI) [2,3]. Although many patients have localized or medically manageable disease, delayed recognition of DNI may lead to airway compromise, descending mediastinitis, vascular complications, sepsis, or death [4,5,6]. DNI involves the potential spaces and fascial planes of the neck, and extension into these compartments influences imaging, consultation, disposition, and procedural planning.

Initial differentiation of localized disease from deep-space involvement is difficult because symptoms and examination findings are nonspecific and overlap across conditions [5,7]. History-taking and examination in the ED may also be constrained by severe pain, trismus, communication difficulty, time pressure, and diagnostic uncertainty [8,9,10]. Contrast-enhanced neck computed tomography (CT) identifies deep-space inflammation and abscess formation, defines anatomical extent, and evaluates relationships to airway and vascular structures [4,11,12]. However, CT is selectively obtained according to clinical judgment rather than applied uniformly.

Routine inflammatory markers may provide such information. Absolute neutrophil count (ANC) reflects the circulating neutrophil response, C-reactive protein (CRP) reflects hepatic acute-phase signaling, and erythrocyte sedimentation rate (ESR) provides an indirect measure of systemic inflammation [13,14,15]. Derived indices—including the neutrophil-to-lymphocyte ratio (NLR), platelet-to-lymphocyte ratio (PLR), systemic immune-inflammation index (SII), systemic inflammation response index (SIRI), aggregate index of systemic inflammation (AISI), CRP-to-albumin ratio (CAR), neutrophil percentage-to-albumin ratio (NPAR), CRP-Albumin-Lymphocyte (CALLY) Index, and Hemoglobin, Albumin, Lymphocyte, and Platelet (HALP) score—combine routinely measured cellular or acute-phase components [16,17,18,19,20,21,22,23,24].

Previous DNI biomarker studies have mainly addressed prognosis, complications, drainage, hospital course, or airway management among patients with established infection [25,26,27,28,29,30,31]. Less is known about laboratory patterns among the broader population of ED patients presenting with throat or neck symptoms before deep-space infection has been established. This study therefore evaluated which routinely available conventional and derived inflammatory markers were associated with DNI in a symptom-based ED cohort. A secondary analysis restricted to patients undergoing contrast-enhanced neck CT assessed whether the principal findings were reproduced using an anatomically defined outcome.

## 2. Materials and Methods

### 2.1. Study Design and Setting

This single-center retrospective observational study was conducted at a tertiary regional emergency medical center from 1 January 2021 through 31 May 2026. The Institutional Review Board of Inha University Hospital approved the study (2026-07-010) and waived informed consent because de-identified retrospective data were used. Structured demographics, vital signs, laboratory results, and encounter diagnoses were extracted from the institutional Clinical Data Warehouse. Chief-complaint text, symptom-onset date and time, and CT report classification were obtained from the electronic medical record using a prespecified case-report form. The study was reported in accordance with the Strengthening the Reporting of Observational Studies in Epidemiology (STROBE) statement; the completed checklist is provided as Supplementary Material.

### 2.2. Study Participants and Eligibility Criteria

Adults aged ≥18 years were eligible when the standardized ED chief-complaint field contained a prespecified throat- or neck-related presentation compatible with infectious or inflammatory disease: sore throat or throat pain, odynophagia or dysphagia, nonposterior neck pain or discomfort, neck or submandibular swelling, dyspnea or stridor, or voice change or hoarseness. The ED system requires selection from a standardized chief-complaint list; synonymous complaint terms were grouped during data extraction rather than identified by unrestricted free-text spelling. Exclusions were traumatic or musculoskeletal presentations, isolated foreign body without target infection, postoperative or iatrogenic presentations, and tumor-related or indeterminate presentations. Postoperative or iatrogenic presentations were identified from encounter/procedural context and record review, whereas tumor-related presentations were identified using medical history, index diagnoses, and clinical documentation. The unit of analysis was the patient; when more than one eligible ED encounter occurred, the first eligible encounter was retained as the index visit.

Because 7-day institutional follow-up was available for only a subset of index-negative patients, delayed diagnoses were described separately rather than used to redefine the primary outcome.

### 2.3. Outcome Definition and Ascertainment

The primary outcome was DNI confirmed during the index episode using prespecified diagnostic evidence. Qualifying evidence included contrast-enhanced neck CT demonstrating infection of a nonperitonsillar deep neck space; endoscopic or fibroscopic findings documenting compatible deep laryngopharyngeal involvement; or ultrasonographic demonstration of a deep-space collection with aspiration or drainage when applicable. For transferred patients who had undergone CT at a referring institution and presented with an external CT report documenting the diagnosis, the outside CT findings were recorded as clinical context. The CT-defined subgroup required study-institution CT images available for protocolized adjudication; when outside images were unavailable, external CT history alone was not treated as study CT confirmation, and primary-outcome confirmation required qualifying in-house endoscopic/fibroscopic or ultrasonographic/procedural evidence. For cases without study-institution CT, anatomical regions were abstracted from the locations documented by the treating otolaryngology team during endoscopic, ultrasonographic, or procedural assessment and were used descriptively rather than as CT-equivalent fascial-plane adjudication.

For CT-evaluated patients, CT-defined DNI included a nonperitonsillar deep-space abscess, cellulitis, phlegmon, or inflammatory change, as well as peritonsillar or intratonsillar infection extending into a nonperitonsillar deep neck space. Isolated peritonsillar or intratonsillar abscess without deep-space extension was classified as no CT-defined DNI. Anatomical spaces were coded individually and included the masticator, submandibular, sublingual, parotid, parapharyngeal, retropharyngeal, danger, carotid, prevertebral, anterior visceral/pretracheal, preepiglottic, and paraglottic spaces; multiple-space involvement was permitted. CT-positive cases were also categorized as abscess or non-abscess DNI for phenotype-specific analyses.

To assess possible delayed recognition among patients without confirmed DNI during the index episode, subsequent encounters within the same institution were reviewed for 7 days after the index visit. Potential delayed DNI diagnoses were verified against the corresponding clinical record. Follow-up outside the study institution was not available.

### 2.4. CT Selection and Imaging Review

The decision to perform contrast-enhanced neck CT was not protocolized and was ordered at the discretion of the attending emergency physician on the basis of the overall clinical assessment, including presenting symptoms, physical examination, and available laboratory findings. In the CT-evaluated cohort, the laboratory results used in this study were available before the CT decision. CT images were initially reviewed and classified by one board-certified emergency physician according to the prespecified anatomical criteria, and the classification was cross-checked against the formal radiology report. Laboratory values were not used as criteria for outcome classification. When the image-based assessment or its concordance with the radiology report was equivocal, the case was discussed with a second board-certified emergency physician, and the final classification was determined by consensus.

CT morphology was classified separately from the overall DNI outcome. A definite abscess required a discrete fluid collection or other imaging description compatible with a drainable collection, whereas cellulitis, phlegmon, edema, fat stranding, and nonorganized inflammatory change without a definite deep-space collection were categorized as non-abscess DNI. These categories were used only for secondary phenotype analyses and did not alter the primary definition of deep-space infection.

### 2.5. Variables and Laboratory Markers

Collected variables included age, sex, structured chief complaint, medical history, symptom-onset time, CT classification, initial ED vital signs, treatment, and disposition. For laboratory variables, the earliest available value from the index ED evaluation was used when repeated measurements were present. ANC was calculated from WBC count and automated differential percentages. Laboratory methods and reference ranges for the principal hematologic and inflammatory tests did not materially change during the study period. Documented prescriptions for antibacterial agents and systemic oral or injectable corticosteroids issued within the institutional system during the 7 days preceding the index ED visit were also captured. Systemic corticosteroid exposure was identified using drug-level glucocorticoid codes; medication prescribed outside the institution could not be ascertained. Symptom duration was calculated from the documented symptom-onset date/time to ED arrival. Approximate day-level onset histories produced heaping at whole-day multiples; values were retained as documented and were neither rounded nor top-coded at 72 h.

Derived markers were calculated as follows: NLR, neutrophils/lymphocytes [16]; PLR, platelets/lymphocytes [17]; SII, platelets × neutrophils/lymphocytes [18]; SIRI, neutrophils × monocytes/lymphocytes [19]; AISI, neutrophils × monocytes × platelets/lymphocytes [20]; CAR, CRP/albumin [21]; and NPAR, neutrophil percentage/albumin [22]. The CALLY index was calculated from albumin, absolute lymphocyte count, and CRP, and the HALP score from hemoglobin, albumin, absolute lymphocyte count, and platelet count according to published formulas [23,24].

### 2.6. Statistical Analysis

Continuous variables are presented as medians with interquartile ranges (IQRs), and categorical variables as counts and percentages. Between-group comparisons used the Mann–Whitney U test and Pearson chi-square or two-sided Fisher exact test, as appropriate. The primary study population comprised the full eligible symptom-based cohort. Individual analyses were performed using analysis-specific complete cases according to the variables required for each model; therefore, sample sizes varied across analyses. Patients with missing laboratory measurements were retained in the study cohort but excluded from analyses requiring those measurements. Because laboratory missingness largely reflected selective test ordering during routine clinical care, missing values were not imputed. For conventional inflammatory markers, functional form was assessed by comparing original and log2-transformed specifications using model fit and inspection for nonlinearity; ANC and WBC were retained on their original scales, whereas CRP and ESR were log2-transformed. Because ANC and WBC provide overlapping leukocyte information, they were not entered simultaneously. Two parallel conventional-marker logistic regression models included age, sex, log2 (CRP), log2 (ESR), and either ANC or WBC. Odds ratios were reported per 5 × 10^9^/L increase in ANC or WBC and per two-fold increase in CRP or ESR.

The laboratory pair carried forward to the incremental analysis was selected from the conventional-marker analysis on the basis of relative model fit and consistency of adjusted association, while avoiding redundant leukocyte predictors. Candidate variables for the structured clinical baseline model were drawn from non-laboratory characteristics available at the initial ED assessment that differed according to confirmed DNI status in the baseline comparisons. Very sparse clinical findings and obviously redundant measures were not carried forward to reduce model instability and collinearity. The resulting clinical model included age, sex, fever, sore throat or odynophagia, neck pain, log2-transformed symptom duration, temperature, systolic blood pressure, heart rate, and respiratory rate. Because both the clinical-variable set and the laboratory-marker pair were data-informed using the same cohort, the incremental analysis was considered internally exploratory rather than a prespecified prediction model. Clinical-variable and laboratory-marker selection was performed before cross-validation and was not repeated within individual training folds; the cross-validated performance estimates therefore represent internal exploratory performance conditional on the selected feature set and may remain optimistic. ANC and CRP were then added jointly using the same functional forms as in the conventional-marker analysis. Incremental performance was assessed primarily using 10-fold cross-validated AUC, the bootstrap 95% confidence interval for the change in AUC, and a likelihood-ratio test; Brier score and calibration slope were examined as supportive measures. Sensitivity analyses examined three assumptions: reclassification of the 27 clinically verified DNI diagnoses occurring within 7 days after an index-negative visit as positive events; a broader outcome that also classified the 103 isolated peritonsillar or intratonsillar abscess cases without nonperitonsillar extension as positive events; and inverse-probability-of-complete-case weighting to address clinically selective model inclusion. The weighting model included age, sex, fever, sore throat or odynophagia, neck pain, and CT-evaluation status. Conventional and derived inflammatory markers were also evaluated individually in age- and sex-adjusted exploratory models, with discrimination summarized by AUC; formal paired AUC comparisons are reported in the Supplementary Materials.

Secondary analyses were restricted to patients who underwent contrast-enhanced neck CT, providing a uniform imaging-based anatomical outcome. The CT analysis repeated the parallel conventional-marker and incremental-value analyses and additionally evaluated anatomical distribution and abscess versus non-abscess phenotypes. Among CT-defined DNI cases, exploratory phenotype analyses compared definite abscess with non-abscess DNI using separate age- and sex-adjusted logistic regression models for each marker; WBC and ANC were reported per 5 × 10^9^/L increase, and CRP and ESR per two-fold increase. Analyses used complete cases for the variables included in each model. Model-specific sample sizes and event counts are reported with the corresponding results. Two-sided *p* < 0.05 was considered statistically significant. Analyses used Python 3.13, SciPy 1.17, patsy 1.0, statsmodels 0.14.6, and scikit-learn 1.8.0.

## 3. Results

### 3.1. Study Population

**Figure 1:**
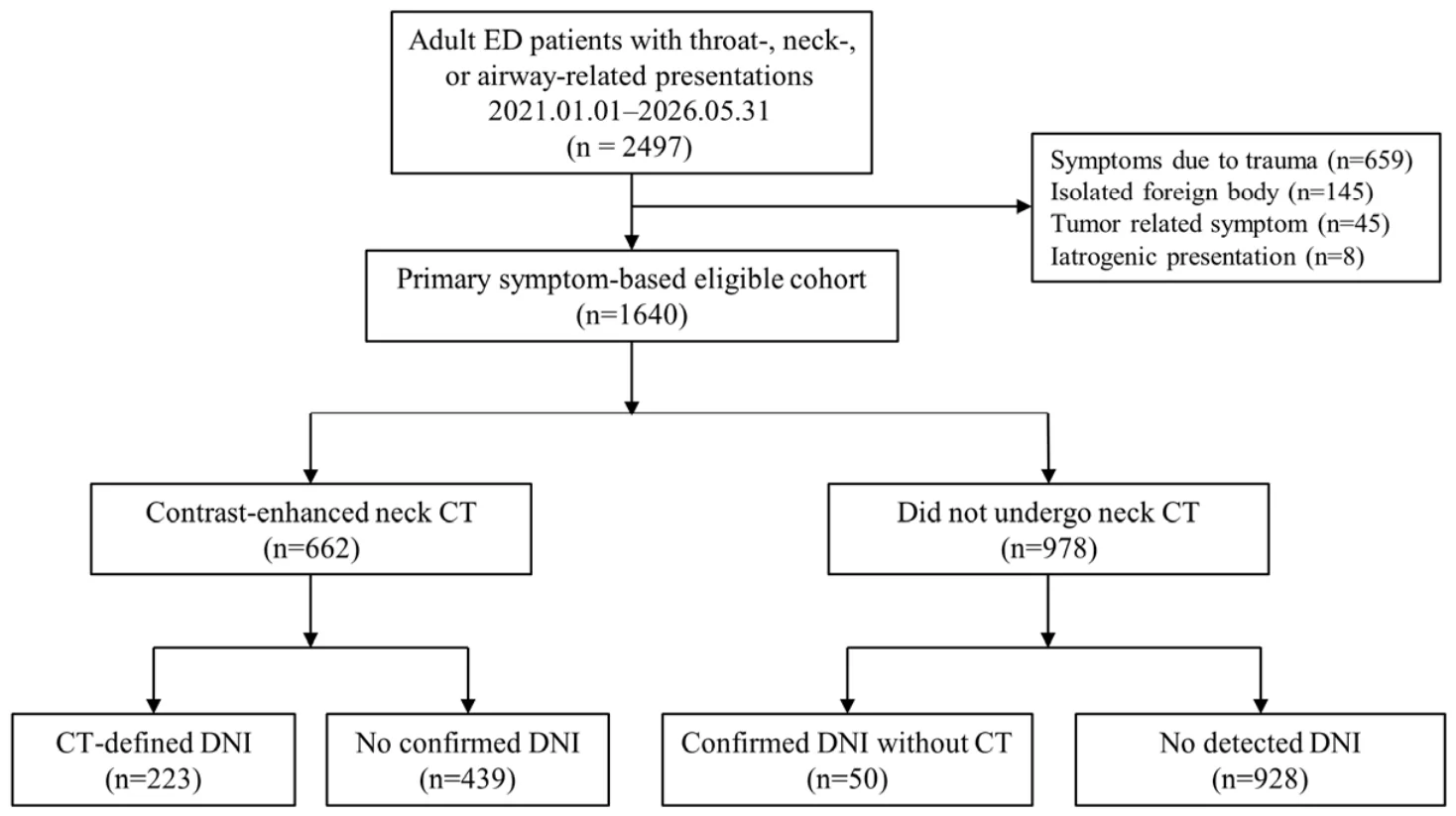
Study flow and outcome ascertainment. The primary analysis included the full symptom-based ED cohort. The CT-defined subgroup was analyzed secondarily using a uniform imaging-based anatomical reference. CT status in the flow diagram refers to study-institution contrast-enhanced neck CT available for protocolized adjudication. CT, computed tomography; ED, emergency department; DNI, deep neck infection.

After application of the prespecified eligibility criteria, 1640 patients formed the primary symptom-based cohort (Figure 1). During the index episode, 273 patients (16.6%) met the predefined criteria for confirmed DNI and 1367 (83.4%) did not have confirmed DNI. Study-institution contrast-enhanced neck CT was performed in 662 patients, of whom 223 had CT-defined DNI and 439 did not; 978 patients were evaluated without study-institution neck CT. Among the 50 confirmed DNI cases in the latter group, all underwent otolaryngology endoscopic/fibroscopic evaluation at the study institution. Thirty-five (70.0%) presented with an external CT report documenting DNI before transfer, but the external images could not be accessed through the institutional system and were unavailable for study-specific adjudication. Primary in-house confirmation was based on endoscopy/fibroscopy in 44 cases (88.0%) and ultrasonography with aspiration/drainage in 6 (12.0%); 11 cases (22.0%) had additional procedural or operative corroboration. ENT-documented anatomical regions were non-mutually exclusive and were most commonly parapharyngeal (25 [50.0%]) and retropharyngeal/posterior pharyngeal (16 [32.0%]) (Supplementary Table S1A,B).

Among the 1367 patients without confirmed DNI at the index visit, 574 (42.0%) had a subsequent institutional record within 7 days. A subsequent DNI diagnosis was verified in 27 patients (2.0% of all index-negative patients and 4.7% of those with an institutional record during the 7-day window). This follow-up was limited to the study institution and therefore did not provide complete negative verification. These 27 patients were retained as index-negative in the primary analysis because they did not meet the index-episode outcome definition; a sensitivity analysis reclassifying all 27 as positive events is reported in Supplementary Table S2.

### 3.2. Baseline Characteristics

Patients with confirmed DNI were older and more often male than those without confirmed DNI (Table 1). Symptom duration was longer in the DNI group (median, 46.8 vs. 25.0 h; *p* < 0.001), and neck pain and neck/submandibular swelling were more common. Fever did not differ significantly between patients without and with confirmed DNI (18.5% vs. 16.5%; *p* = 0.428). WBC count, ANC, ESR, and CRP were higher, whereas albumin was lower. NLR, PLR, SII, SIRI, AISI, CAR, and NPAR were higher in the DNI group, whereas CALLY and HALP were lower. Recorded antibiotic and systemic corticosteroid prescriptions within the preceding 7 days were uncommon (37 [2.3%] and 24 [1.5%], respectively) and did not differ significantly according to DNI status. Otolaryngology consultation was common among patients with confirmed DNI, consistent with its role in the diagnostic ascertainment pathway; hospital admission and drainage were also more frequent.

The CT-evaluated subgroup comprised 662 patients, including 223 (33.7%) with CT-defined DNI. Compared with the 978 patients evaluated without study-institution neck CT, CT-evaluated patients were older, had longer symptom duration, and had higher WBC, ANC, CRP, and ESR values (Supplementary Table S3A). Recorded otolaryngology consultation occurred in 535 of 662 CT-evaluated patients (80.8%) versus 151 of 978 patients without study-institution CT (15.4%), consistent with a clinically selected higher-risk spectrum entering the imaging-defined analyses. Supplementary Table S3B shows anatomical distribution of CT-defined DNI. Among the 223 CT-defined DNI cases, 72 (32.3%) were classified as definite abscess and 151 (67.7%) as non-abscess DNI (Supplementary Table S4A). Among patients with CT-defined DNI, definite abscess was associated with higher WBC and ANC than non-abscess DNI, and both associations remained after adjustment for age and sex, whereas CRP and ESR did not clearly distinguish the two morphological phenotypes (Supplementary Table S4B).

### 3.3. Conventional-Marker Association Analysis

Functional-form assessment supported raw-linear ANC and WBC and log2-transformed CRP and ESR. Of the 1640 patients in the primary symptom-based cohort, 1152 had complete data for the variables required in the parallel conventional-marker models, including 266 patients with confirmed DNI (Table 2A). In the ANC-based model, ANC remained associated with confirmed DNI (aOR per 5 × 10^9^/L, 1.51; 95% CI, 1.27–1.80; *p* < 0.001), as did CRP (aOR per two-fold increase, 1.33; 95% CI, 1.19–1.48; *p* < 0.001), whereas ESR did not reach statistical significance (aOR per two-fold increase, 1.14; 95% CI, 0.99–1.30; *p* = 0.066). In the WBC-based model, WBC (aOR per 5 × 10^9^/L, 1.38; 95% CI, 1.17–1.62; *p* < 0.001) and CRP (aOR, 1.36; 95% CI, 1.23–1.52; *p* < 0.001) remained associated, whereas ESR did not (aOR, 1.11; 95% CI, 0.97–1.27; *p* = 0.133). The ANC-based model had the lower AIC (1083.89 vs. 1089.84).

In the CT-evaluated subgroup, the same parallel conventional-marker models included 607 complete cases with 217 CT-defined DNI events (Table 2B). In the ANC-based model, ANC (aOR per 5 × 10^9^/L, 1.35; 95% CI, 1.11–1.65; *p* = 0.003) and CRP (aOR per two-fold increase, 1.18; 95% CI, 1.05–1.33; *p* = 0.007) were associated with CT-defined DNI, whereas ESR was not (aOR, 1.02; 95% CI, 0.87–1.19; *p* = 0.838). In the WBC-based model, WBC did not reach conventional statistical significance (aOR per 5 × 10^9^/L, 1.18; 95% CI, 0.99–1.40; *p* = 0.058), whereas CRP remained associated (aOR, 1.22; 95% CI, 1.09–1.38; *p* < 0.001) and ESR did not (aOR, 0.99; 95% CI, 0.85–1.15; *p* = 0.866). The ANC-based model again had the lower AIC (758.67 vs. 764.23).

### 3.4. Clinical-Model Performance and Incremental Value of ANC and CRP

For the incremental analysis, which did not require ESR measurement, 1166 of the 1640 patients had complete data for all clinical variables, ANC, and CRP, including 268 patients with confirmed DNI. Compared with these complete cases, the 474 patients excluded because of at least one missing model variable had substantially lower frequencies of confirmed DNI (1.1% vs. 23.0%) and CT evaluation (8.2% vs. 53.4%), consistent with clinically selective testing (Supplementary Table S5). ANC was carried forward rather than WBC because the two markers convey overlapping leukocyte information and the ANC-based conventional model had the lower AIC in the primary cohort; CRP was retained because it remained associated in both primary conventional-marker models, whereas ESR did not. In the primary symptom-based cohort, the structured clinical model yielded a 10-fold cross-validated AUC of 0.644, which increased to 0.788 after addition of ANC and CRP (ΔAUC, 0.144; bootstrap 95% CI, 0.109–0.179; likelihood-ratio *p* < 0.001). The Brier score improved from 0.169 to 0.143, and the calibration slope moved from 0.843 to 0.921. In the augmented model, ANC (aOR per 5 × 10^9^/L, 1.49; 95% CI, 1.25–1.79; *p* < 0.001) and CRP (aOR per two-fold increase, 1.51; 95% CI, 1.37–1.65; *p* < 0.001) remained associated with DNI. The incremental improvement was similar when the 27 verified delayed DNI diagnoses were reclassified as positive (ΔAUC, 0.139; 95% CI, 0.104–0.172), when isolated peritonsillar/intratonsillar abscesses were included in a broader positive outcome (ΔAUC, 0.148; 95% CI, 0.116–0.180), and with inverse-probability-of-complete-case weighting (ΔAUC, 0.139; 95% CI, 0.106–0.174) (Supplementary Table S2).

In the CT-evaluated subgroup, 623 complete cases with 219 CT-defined DNI events were included (Table 3B). The structured clinical model yielded a 10-fold cross-validated AUC of 0.562. After addition of ANC and CRP, the AUC increased to 0.685 (ΔAUC, 0.123; bootstrap 95% CI, 0.077–0.167), and the likelihood-ratio test again favored the augmented model (*p* < 0.001). These CT-subgroup results were treated as secondary consistency analyses and did not determine marker selection. The calibration slope moved closer to 1 after addition of ANC and CRP, from 0.843 to 0.921 in the primary cohort and from 0.490 to 0.780 in the CT subgroup; calibration therefore improved with laboratory augmentation but remained imperfect, particularly in the CT subgroup.

### 3.5. Marker-Specific Analyses

In separate age- and sex-adjusted single-marker analyses (Table 4), WBC, ANC, CRP, ESR, and albumin were each associated with confirmed DNI. Their univariable AUCs were 0.719, 0.735, 0.742, 0.672, and 0.610, respectively. Lower albumin was associated with confirmed DNI (aOR per 0.5 g/dL decrease, 1.57; 95% CI, 1.33–1.85; *p* < 0.001), but its discrimination was weaker than that of ANC or CRP. Among the derived indices, AUCs were 0.709 for NLR, 0.575 for PLR, 0.699 for SII, 0.732 for SIRI, 0.722 for AISI, 0.742 for CAR, 0.715 for NPAR, 0.729 for CALLY, and 0.580 for HALP. Formal paired comparisons using matched observations are provided in Supplementary Table S6; no composite index showed a clear discriminatory advantage over the simpler conventional markers.

## 4. Discussion

In this symptom-based ED cohort, conventional inflammatory markers provided clinically relevant information beyond the initial clinical assessment, with ANC and CRP emerging as the most consistent markers across the principal analyses. Importantly, the clinical contribution of these findings lies not simply in the expected association of ANC and CRP with infection, but in their incremental value beyond demographics, presenting symptoms, vital signs, and symptom duration. When ANC and CRP were added to the structured clinical model, the 10-fold cross-validated AUC increased from 0.644 to 0.788 (ΔAUC, 0.144). Similar increments were observed after alternative outcome definitions and inverse-probability weighting for complete-case selection, indicating that the incremental signal was not dependent on a single outcome boundary or the unweighted complete-case composition. A similar incremental pattern was observed in the CT-defined secondary analysis, supporting consistency across different outcome-ascertainment frameworks. In contrast, more complex derived inflammatory indices did not clearly outperform conventional markers, suggesting that much of the useful laboratory information may already be captured by simple, routinely available measurements.

The change in study frame is clinically relevant. Most previous biomarker studies began with patients who already had established DNI and evaluated prognosis, drainage, hospitalization, airway management, or other aspects of clinical course [25,26,27,28,29,30,31]. Our primary population instead began with adults presenting to the ED with throat or neck symptoms, before DNI had been established. The 16.6% overall DNI prevalence therefore reflects a broader clinical sampling frame than the 33.7% prevalence in the CT-selected subgroup and more closely represents the point at which laboratory information is first considered alongside symptoms, examination findings, and vital signs.

The consistency of ANC is biologically plausible. Neutrophils are central effector cells of innate antibacterial immunity and can increase rapidly through redistribution within circulating granulocyte pools and mobilization from bone marrow [13,32]. Deep-space extension may increase local inflammatory burden and neutrophil recruitment, producing a measurable systemic response. ANC directly quantifies this cellular component, whereas total WBC includes several leukocyte lineages unrelated to acute neutrophilic inflammation. This may partly explain why the ANC-based specification showed better fit than the otherwise parallel WBC-based model in both the primary and CT-defined analyses.

CRP provided complementary information in the overall ED cohort. CRP reflects cytokine-driven hepatic acute-phase signaling and generally rises several hours after inflammatory stimulation [15]. In the primary cohort, patients with DNI had longer symptom duration, which may have contributed to the higher CRP concentrations observed at presentation. Nevertheless, CRP retained an adjusted association in both parallel conventional-marker models and also remained associated in the CT-defined subgroup. The smaller effect estimate in the CT subgroup may reflect the narrower, clinically selected inflammatory spectrum among patients already judged to require CT rather than a fundamentally different biological relationship. Taken together, the persistence of both ANC and CRP suggests that cellular and acute-phase responses provide overlapping but nonidentical information about the presence of deep-space infection.

ESR showed a different pattern. Although ESR was higher in patients with DNI and was associated with DNI in marker-specific analysis, it did not retain a clear association when the leukocyte measure and CRP were considered simultaneously. ESR responds more slowly than CRP and is influenced by age, sex, anemia, erythrocyte properties, fibrinogen, and immunoglobulins [14,33]. Its elevation may therefore reflect a broader inflammatory milieu while providing less distinct information about the acute infectious process encountered during ED evaluation.

The derived-index findings reinforce this interpretation. NLR, SII, SIRI, AISI, and NPAR all incorporate neutrophil-related information [16,18,19,20,22], while CAR directly incorporates CRP and albumin and CALLY and HALP combine several routinely measured hematologic or inflammatory components [21,23,24]. Their associations therefore partly re-express signals already present in simpler laboratory measurements rather than introducing entirely independent biological information. This likely explains why several composite indices were associated with DNI, yet none showed a clear discriminatory advantage over ANC or CRP in matched comparisons. Mathematical combination of correlated inflammatory components may increase apparent complexity without adding commensurate new information at the initial ED assessment.

The CT-defined analysis adds an important anatomical perspective. The persistence of ANC and CRP associations when the outcome was restricted to radiographically defined deep-space involvement argues that the primary findings were not solely a consequence of clinical diagnostic labeling. At the same time, discrimination was lower in the CT subgroup. This is not unexpected because CT was preferentially obtained in patients who were already clinically more concerning and who had higher inflammatory-marker values, producing restriction of range and creating the possibility of collider stratification through conditioning on CT performance. These selection mechanisms may have altered marker–outcome associations and reduced separation between cases and controls within the CT-selected spectrum. Thus, the CT analysis supports the consistency of the marker signal while also showing why these markers are unlikely to replace anatomical imaging once clinical suspicion is sufficiently high to prompt CT.

The discrimination analyses help define the clinical meaning of these associations. Addition of ANC and CRP improved discrimination beyond routinely available structured clinical characteristics in the full symptom-based cohort, but performance remained only moderate in the CT-defined subgroup. These findings suggest that inflammatory markers may refine, rather than replace, clinical assessment of the likelihood of deep-space involvement. In patients with suspected airway compromise, airway stabilization takes precedence over any laboratory or imaging evaluation; likewise, when deep-neck anatomy must be defined, contrast-enhanced CT is indispensable [11,12] and beyond what laboratory markers can provide. However, when disposition depends on information from clinical assessment and laboratory findings—particularly when examination is limited by pain, trismus, dysphonia, or limited cooperation—clinical assessment alone may underestimate the presence or extent of deep-space disease [34]. ANC and CRP may therefore be most useful as adjuncts to pre-imaging risk estimation in this setting, although they cannot identify the involved anatomical space or establish the presence of a drainable collection. Accordingly, no threshold or rule for obtaining or omitting CT was derived in this study.

This study has several limitations. First, its retrospective single-center design limits generalizability and permits residual confounding and documentation error. Second, outcome verification was not uniform across the full symptom-based cohort because CT and otolaryngology evaluation were clinically selected. Incomplete institutional follow-up and the inability to capture care received elsewhere may have resulted in missed DNI among index-negative patients and partial verification bias. Some transferred patients presented with external CT reports documenting DNI, but the external image data could not be accessed through the institutional system at the time of data collection and were therefore unavailable for study-specific adjudication; these cases consequently depended on qualifying in-house endoscopic, ultrasonographic, or procedural evidence. Although the CT-restricted analysis provided a more anatomically uniform reference, it remained subject to clinical selection for imaging. Third, laboratory testing was also clinically selective, and complete-case analyses therefore reflected nonrandom testing patterns. Inverse-probability-of-complete-case weighting produced a similar incremental AUC difference, but this approach cannot eliminate selection related to unmeasured determinants of testing. Fourth, selection of the clinical-variable set and ANC/CRP pair was informed by the same analytic cohort and was not repeated within individual cross-validation training folds; consequently, the cross-validated performance estimates may remain optimistic and should be regarded as internally exploratory. Fifth, symptom-onset timing was retrospectively documented and showed heaping at whole-day intervals, reflecting limited temporal precision for some histories. Log2 transformation reduced the influence of large absolute timing differences at longer durations, but residual measurement imprecision remains possible. Sixth, CT findings were initially classified by one emergency physician with reference to the formal radiology report, and second-physician consensus review was reserved for equivocal cases; formal interobserver agreement was therefore unavailable. In addition, contrast-enhanced CT does not perfectly distinguish abscess from cellulitis or phlegmon, which should be considered when interpreting the exploratory morphology analyses. Seventh, inflammatory marker levels may have been influenced by prior treatment, symptom duration, underlying hematologic or inflammatory conditions, infection source, and other unmeasured factors. Although in-system antibiotic and corticosteroid prescriptions were captured, treatment received elsewhere and the exact timing of therapy relative to laboratory sampling were unavailable. Finally, this was an association and incremental-information study rather than a diagnostic decision-rule study; prospective validation with standardized outcome verification would be required before clinical thresholds or imaging decisions could be considered.

## 5. Conclusions

Among adults presenting to the ED with throat or neck symptoms, ANC and CRP provided complementary inflammatory information beyond structured clinical assessment, with ANC showing the most consistent association across the overall and CT-defined analyses. Their addition improved discrimination for confirmed DNI, whereas more complex derived inflammatory indices offered no clear advantage over conventional markers. Routinely available inflammatory markers may therefore serve as useful adjuncts during early assessment for DNI, but should be interpreted within the broader clinical and imaging context rather than as stand-alone diagnostic criteria.

## Figures and Tables

**Table 1:** Baseline characteristics of the primary symptom-based cohort according to confirmed DNI.

Variable	Overall (*n* = 1640)	No Confirmed DNI (*n* = 1367)	Confirmed DNI (*n* = 273)	*p* Value
Demographic characteristics				
Age, years	37.0 (28.0–55.0)	35.0 (27.0–52.0)	45.0 (32.0–61.0)	<0.001
Male sex	817 (49.8)	645 (47.2)	172 (63.0)	<0.001
Medical history				
Hypertension	222 (13.5)	177 (12.9)	45 (16.5)	0.129
Diabetes mellitus	146 (8.9)	118 (8.6)	28 (10.3)	0.465
Dyslipidemia	64 (3.9)	51 (3.7)	13 (4.8)	0.433
Malignancy	61 (3.7)	48 (3.5)	13 (4.8)	0.302
Clinical presentation				
Time from symptom onset to ED arrival, h	27.4 (4.5–72.0)	25.0 (2.2–72.0)	46.8 (20.8–72.0)	<0.001
Fever	298 (18.2)	253 (18.5)	45 (16.5)	0.428
Sore throat or odynophagia	1242 (75.7)	1060 (77.5)	182 (66.7)	<0.001
Neck pain	393 (24.0)	305 (22.3)	88 (32.2)	<0.001
Neck or submandibular swelling	16 (1.0)	7 (0.5)	9 (3.3)	<0.001
Dyspnea or stridor	15 (0.9)	10 (0.7)	5 (1.8)	0.081
Voice change or hoarseness	4 (0.2)	2 (0.1)	2 (0.7)	0.131
Initial vital signs				
Temperature, degrees C	36.9 (36.5–37.5)	36.9 (36.5–37.5)	37.1 (36.6–37.7)	<0.001
Systolic blood pressure, mmHg	132.0 (120.0–148.0)	131.0 (119.0–147.0)	138.0 (124.0–153.0)	<0.001
Diastolic blood pressure, mmHg	80.0 (71.0–90.0)	80.0 (70.0–89.0)	83.0 (75.0–92.0)	0.002
Heart rate, /min	90.0 (79.0–103.0)	90.0 (79.0–102.0)	92.0 (84.0–106.0)	0.002
Respiratory rate, /min	18.0 (18.0–20.0)	18.0 (18.0–18.0)	18.0 (18.0–20.0)	<0.001
Oxygen saturation, %	98.0 (97.0–99.0)	98.0 (97.0–99.0)	98.0 (97.0–99.0)	0.010
Initial laboratory findings				
WBC count, ×10^9^/L	10.31 (7.79–13.56)	9.20 (6.92–12.43)	13.02 (9.91–16.43)	<0.001
ANC, ×10^9^/L	7.30 (4.92–10.94)	6.57 (4.41–9.64)	10.50 (7.84–13.32)	<0.001
Platelet count, ×10^9^/L	227.0 (190.0–269.0)	227.0 (190.0–269.0)	223.0 (188.0–270.0)	0.494
ESR, mm/h	16.0 (6.0–30.0)	14.0 (5.0–27.0)	28.0 (13.0–43.0)	<0.001
CRP, mg/dL	2.57 (0.61–8.33)	1.81 (0.42–5.37)	6.71 (3.12–14.68)	<0.001
Albumin, g/dL	4.20 (3.90–4.40)	4.20 (4.00–4.40)	4.00 (3.80–4.30)	<0.001
Derived inflammatory indices				
NLR	5.06 (2.81–8.89)	4.53 (2.49–7.87)	8.09 (5.35–12.89)	<0.001
PLR	158.37 (117.42–218.39)	153.56 (114.77–215.64)	179.26 (128.95–230.51)	<0.001
SII	1201.33 (653.80–1980.20)	1054.89 (551.69–1722.19)	1897.90 (1133.90–2824.17)	<0.001
SIRI	2.82 (1.22–5.57)	2.24 (0.98–4.68)	5.33 (2.81–9.25)	<0.001
AISI	633.13 (286.67–1232.25)	510.92 (235.93–1042.48)	1185.93 (639.31–2047.86)	<0.001
CAR	0.62 (0.16–1.89)	0.44 (0.10–1.31)	1.70 (0.76–3.71)	<0.001
NPAR	18.10 (15.72–20.35)	17.48 (14.90–19.68)	20.05 (17.86–22.22)	<0.001
CALLY	1.91 (0.61–8.33)	2.58 (0.81–11.98)	0.76 (0.32–2.03)	<0.001
HALP	35.57 (24.27–51.09)	36.82 (24.86–52.28)	30.85 (23.26–44.66)	<0.001
Clinical course and management				
Antibiotic prescription within previous 7 days	37 (2.3)	35 (2.6)	2 (0.7)	0.073
Corticosteroid prescription within previous 7 days	24 (1.5)	21 (1.5)	3 (1.1)	0.785
ENT consultation	686 (41.8)	423 (30.9)	263 (96.3)	<0.001
Hospital admission	414 (25.2)	218 (15.9)	196 (71.8)	<0.001
Confirmed drainage procedure	62 (3.8)	16 (1.2)	46 (16.8)	<0.001

Values are median (interquartile range) or *n* (%). Continuous variables were compared using the Mann–Whitney U test; categorical variables using the chi-square or Fisher exact test. Confirmed drainage includes only chart-verified aspiration, incision and drainage, or operative drainage. DNI, deep neck infection; WBC, white blood cell count; ANC, absolute neutrophil count; ESR, erythrocyte sedimentation rate; CRP, C-reactive protein; NLR, neutrophil-to-lymphocyte ratio; PLR, platelet-to-lymphocyte ratio; SII, systemic immune-inflammation index; SIRI, systemic inflammation response index; AISI, aggregate index of systemic inflammation; CAR, CRP-to-albumin ratio; NPAR, neutrophil percentage-to-albumin ratio; CALLY, CRP-Albumin-Lymphocyte Index; HALP, Hemoglobin, Albumin, Lymphocyte, and Platelet score.

**Table 2:** Conventional-marker association models.

(**A**) Primary symptom-based cohort.			
**Predictor**	**Unit**	**aOR (95% CI)**	***p*** **Value**
***ANC-based model***			
Age	per 10 years	1.04 (0.96–1.14)	0.318
Male sex	male vs. female	1.64 (1.20–2.25)	0.002
ANC	per 5 × 10^9^/L	1.51 (1.27–1.80)	<0.001
CRP	two-fold increase	1.33 (1.19–1.48)	<0.001
ESR	two-fold increase	1.14 (0.99–1.30)	0.066
*AIC = 1083.89*			
***WBC-based model***			
Age	per 10 years	1.05 (0.97–1.14)	0.231
Male sex	male vs. female	1.58 (1.16–2.17)	0.004
WBC	per 5 × 10^9^/L	1.38 (1.17–1.62)	<0.001
CRP	two-fold increase	1.36 (1.23–1.52)	<0.001
ESR	two-fold increase	1.11 (0.97–1.27)	0.133
*AIC = 1089.84*			
(**B**) CT-evaluated cohort.			
**Predictor**	**Unit**	**aOR (95% CI)**	***p*** **Value**
***ANC-based model***			
Age	per 10 years	1.11 (1.00–1.23)	0.044
Male sex	male vs. female	1.29 (0.89–1.86)	0.181
ANC	per 5 × 10^9^/L	1.35 (1.11–1.65)	0.003
CRP	two-fold increase	1.18 (1.05–1.33)	0.007
ESR	two-fold increase	1.02 (0.87–1.19)	0.838
*AIC = 758.67*			
***WBC-based model***			
Age	per 10 years	1.12 (1.01–1.24)	0.030
Male sex	male vs. female	1.27 (0.88–1.83)	0.210
WBC	per 5 × 10^9^/L	1.18 (0.99–1.40)	0.058
CRP	two-fold increase	1.22 (1.09–1.38)	<0.001
ESR	two-fold increase	0.99 (0.85–1.15)	0.866
*AIC = 764.23*			

(**A**) Complete-case analysis: *n* = 1152, including 266 confirmed DNI events. (**B**): Complete-case analysis: *n* = 607, including 217 CT-defined DNI events. Both models included age, sex, log2 (CRP), and log2 (ESR), with either ANC or WBC. aOR, adjusted odds ratio; CI, confidence interval; ANC, absolute neutrophil count; WBC, white blood cell; CRP, C-reactive protein; ESR, erythrocyte sedimentation rate; DNI, deep neck infection.

**Table 3:** Incremental performance of ANC and CRP beyond structured clinical variables.

(**A**) Primary symptom-based cohort.				
**Model**	***N***	**Events**	**10-Fold CV AUC**	**Model Comparison**
Clinical model only	1166	268	0.644	—
Clinical + ANC + CRP	1166	268	0.788	LRT *p* < 0.001
Increment	—	—	+0.144	ΔAUC 95% CI 0.109–0.179
(**B**) CT-evaluated cohort.				
**Model**	***N***	**Events**	**10-Fold CV AUC**	**Model Comparison**
Clinical model only	623	219	0.562	—
Clinical + ANC + CRP	623	219	0.685	LRT *p* < 0.001
Increment	—	—	+0.123	ΔAUC 95% CI 0.077–0.167

(**A**) The clinical model included age, sex, fever, sore throat/odynophagia, neck pain, log2 (symptom duration + 1), temperature, systolic blood pressure, heart rate, and respiratory rate. ANC and CRP were added jointly using the same functional forms as in the conventional-marker analysis. The same complete-case sample was used for both models (*n* = 1166; DNI events = 268). (**B**): The clinical baseline variables were identical to those used in (**A**). ANC and CRP were added using the same functional forms. The same complete-case CT sample was used for both models (*n* = 623; CT-defined DNI events = 219). CT, computed tomography; CV, cross-validation; AUC, area under the receiver operating characteristic curve; ANC, absolute neutrophil count; CRP, C-reactive protein; LRT, likelihood-ratio test; CI, confidence interval.

**Table 4:** Marker-specific age- and sex-adjusted associations and discrimination in the primary symptom-based cohort.

**Marker**	***N*** **(Events)**	**Adjusted OR (95% CI)**	***p*** **Value**	**AUC**
WBC count	1282 (271)	2.18 (1.83–2.61)	<0.001	0.719
ANC	1281 (271)	2.46 (2.05–2.95)	<0.001	0.735
ESR	1303 (269)	2.56 (2.05–3.21)	<0.001	0.672
CRP	1251 (271)	4.29 (3.31–5.56)	<0.001	0.742
Albumin	1357 (271)	1.57 (1.33–1.85) per 0.5 g/dL decrease	<0.001	0.610
NLR	1281 (271)	1.50 (1.32–1.69)	<0.001	0.709
PLR	1279 (271)	1.24 (1.10–1.41)	<0.001	0.575
SII	1279 (271)	1.51 (1.33–1.71)	<0.001	0.699
SIRI	1281 (271)	1.46 (1.31–1.64)	<0.001	0.732
AISI	1279 (271)	1.36 (1.23–1.50)	<0.001	0.722
CAR	1247 (271)	1.71 (1.52–1.92)	<0.001	0.742
NPAR	1290 (273)	2.40 (1.98–2.90)	<0.001	0.715
CALLY	1173 (269)	3.80 (2.93–4.93)	<0.001	0.729
HALP	1276 (271)	1.50 (1.25–1.78)	<0.001	0.580

Each marker was evaluated in a separate logistic regression model adjusted for age and sex. Odds ratios are reported per observed IQR increase in the modeled marker, except albumin, CALLY, and HALP, for which ORs are reported per observed IQR decrease so that an OR > 1 represents a less favorable inflammatory profile. WBC, ANC, albumin, NLR, PLR, SII, SIRI, AISI, CAR, and NPAR were modeled on their original scales, whereas CRP, ESR, CALLY, and HALP were modeled on log2-transformed scales; therefore, the reported IQRs for CRP, ESR, CALLY, and HALP represent IQR changes on the log2 scale. These marker-specific analyses were exploratory and were not adjusted for multiple comparisons. OR, odds ratio; CI, confidence interval; IQR, interquartile range; WBC, white blood cell count; ANC, absolute neutrophil count; ESR, erythrocyte sedimentation rate; CRP, C-reactive protein; NLR, neutrophil-to-lymphocyte ratio; PLR, platelet-to-lymphocyte ratio; SII, systemic immune-inflammation index; SIRI, systemic inflammation response index; AISI, aggregate index of systemic inflammation; CAR, CRP-to-albumin ratio; NPAR, neutrophil percentage-to-albumin ratio.

## Data Availability

The data presented in this study are available on request from the corresponding author due to patient consent for the use of personal information.

## Supplementary Materials

The following supporting information can be downloaded at: https://www.mdpi.com/article/10.3390/diagnostics16183016/s1, File S1. STROBE Statement—Checklist of items that should be included in reports of cohort studies. Table S1. Outcome ascertainment in the 50 confirmed DNI cases without study-institution CT. (A) Confirmation pathway among confirmed DNI cases without study-institution CT (*n* = 50). (B) ENT-documented anatomical regions among these 50 cases. Table S2. Sensitivity analyses of the incremental value of ANC and CRP. Table S3. (A) CT-evaluated versus patients without study-institution CT. (B) Anatomical distribution. Table S4. Anatomical distribution and abscess versus non-abscess morphology of CT-defined DNI (*n* = 223). (A) Morphology classification. (B) Laboratory markers and age- and sex-adjusted associations with definite abscess. Table S5. Comparison of patients included in and excluded from the primary incremental analysis. Table S6. Comparison of discriminatory performance of derived inflammatory indices with ANC and CRP.

## Abbreviations

AIC	Akaike information criterion
AISI	aggregate index of systemic inflammation
ANC	absolute neutrophil count
aOR	adjusted odds ratio
AUC	area under the receiver operating characteristic curve
CAR	C-reactive protein–to–albumin ratio
CI	confidence interval
CRP	C-reactive protein
CT	computed tomography
CV	cross-validation
DNI	deep neck infection
ED	emergency department
ESR	erythrocyte sedimentation rate
IQR	interquartile range
IRB	Institutional Review Board
NLR	neutrophil-to-lymphocyte ratio
NPAR	neutrophil percentage–to–albumin ratio
OR	odds ratio
PLR	platelet-to-lymphocyte ratio
SII	systemic immune-inflammation index
SIRI	systemic inflammation response index
WBC	white blood cell (count)
