# Supplementary material for: Laboratory Markers Associated with Deep Neck Infection in Emergency Department Patients with Throat or Neck Symptoms
Source: Diagnostics (Basel). 2026 Sep 17;16(18):3016. doi: 10.3390/diagnostics16183016 (PMC13605789; doi:10.3390/diagnostics16183016)
Supplement: Supplementary file 1 [file diagnostics-16-03016-s001.zip › diagnostics-4492460-supplementary File S1.pdf]

STROBE Statement—Checklist of items that should be included in reports of cohort studies

Manuscript: "Laboratory Markers Associated with Deep Neck Infection in Emergency Department Patients with Throat or Neck Symptoms" (Manuscript ID: diagnostics-4492460)

Section	Item No	Recommendation	Location in manuscript / how the item is addressed
Title and abstract	1(a)	Indicate the study's design with a commonly used term in the title or the abstract	The design is stated as a "single-center retrospective cohort" in the Abstract (Methods) and in Section 2.1. (Abstract p.1; Section 2.1)
Title and abstract	1(b)	Provide in the abstract an informative and balanced summary of what was done and what was found	The Abstract follows a structured Background/Methods/Results/Conclusions format and reports the cohort size (n=1,640), the primary outcome (confirmed DNI, 273 patients; 16.6%), the principal result (incremental ΔAUC 0.138; 95% CI 0.104–0.172), and the key limitation ("insufficient for stand-alone diagnostic or imaging decisions"). (p.1)
Background/rationale	2	Explain the scientific background and rationale for the investigation being reported	Section 1 (Introduction), first to third paragraphs: the difficulty of distinguishing DNI from localized disease, the role and selective use of contrast-enhanced CT, and the rationale for routinely available inflammatory markers. (pp.1–2)
Objectives	3	State specific objectives, including any prespecified hypotheses	Introduction, fourth paragraph: evaluation of associations of conventional and derived markers with confirmed DNI in a symptom-based ED cohort, reproduction in the CT-defined subgroup, and incremental value beyond a structured clinical model. (p.2)
Study design	4	Present key elements of study design early in the paper	Section 2.1: single-center retrospective observational cohort; study period January 1, 2021–May 31, 2026. (p.3)
Setting	5	Describe the setting, locations, and relevant dates, including periods of recruitment, exposure, follow-up, and data	Section 2.1: tertiary regional emergency medical center (Inha University Hospital); IRB approval (2026-07-010) with waiver of consent. (p.3; Declarations p.11)

		collection	
Participants	6	Give the eligibility criteria, and the sources and methods of selection of participants	Section 2.2, first paragraph: adults (≥18 years) with prespecified chief-complaint categories (six categories enumerated), operational exclusions (traumatic, isolated foreign body, tumor-related, postoperative/iatrogenic), and unit of analysis (patient; first eligible encounter only). Figure 1 shows the selection flow (2,497 screened → 1,640 included). (p.4)
Variables	7	Clearly define all outcomes, exposures, predictors, potential confounders, and effect modifiers	Section 2.3 (primary outcome: confirmed DNI requiring prespecified objective evidence — CT, endoscopic/fibroscopic evaluation, or ultrasonographic evaluation with aspiration/drainage), Section 2.4 (CT review and morphology classification), Section 2.5 (variables, derived-index formulas, prior antibiotic/corticosteroid definitions). (pp.4–5)
Data sources/measurement	8	For each variable of interest, give sources of data and details of methods of assessment	Section 2.1 (Clinical Data Warehouse and EMR extraction), Section 2.4 (imaging review procedure; laboratory results available before CT decision), Section 2.5 ("initial" values defined as the earliest index-encounter measurement; ANC derived from WBC and automated differential; laboratory methods and reference ranges unchanged during the study period). (pp.3–5)
Bias	9	Describe any efforts to address potential sources of bias	Section 2.3, third paragraph (7-day institutional follow-up to assess delayed recognition), Section 2.6 (covariate adjustment, complete-case analysis, 10-fold cross-validation); Discussion, ninth paragraph (partial verification bias, selection into imaging, restriction of range and collider stratification, with expected direction stated). (pp.4–5, 9–10)
Study size	10	Explain how the study size was arrived at	Retrospective cohort of all consecutive eligible patients during the study period (no a priori sample-size calculation); model-specific complete-case samples and event counts are reported (1,152/266; 1,166/268; 607/217; 623/219). (Sections 2.1, 2.6; Tables 2–4 footnotes)

Quantitative variables	11	Explain how quantitative variables were handled in the analyses	Section 2.6: ANC and WBC on original scale (per 5 × 10^9/L); CRP and ESR log2-transformed; functional-form assessment (restricted cubic splines); marker-specific analyses scaled per IQR. (p.5)
Statistical methods	12(a)	Describe all statistical methods, including those used to control for confounding	Section 2.6, first and second paragraphs: Mann–Whitney U, chi-square/Fisher exact tests; multivariable logistic regression (adjustment for age, sex, and clinical variables); parallel ANC- and WBC-based models; AIC; AUC with likelihood-ratio tests, and calibration (Brier score, calibration slope); software (Python 3.13, SciPy 1.17, statsmodels 0.14.6, scikit-learn). (p.5)
Statistical methods	12(b)	Describe any methods used to examine subgroups and interactions	Secondary analysis restricted to the CT-evaluated subgroup (n=662) and an exploratory abscess-versus-non-abscess phenotype analysis (explicitly labeled exploratory). (Section 2.6, third paragraph; Section 3.5; Supplementary Table S2)
Statistical methods	12(c)	Explain how missing data were addressed	Section 2.6, first paragraph: complete-case analysis; because missingness largely reflected tests not ordered during routine care and is plausibly not random, no imputation was performed; variable-level availability (e.g., ESR n=1,303) and included-versus-excluded patient characteristics are reported (Supplementary Table S3). (p.5)
Statistical methods	12(d)	If applicable, explain how loss to follow-up was addressed	Not applicable in the conventional sense for this retrospective single-encounter cohort; instead, delayed recognition among index-negative patients was assessed through 7-day institutional follow-up (574/1,367 with records, 42.0%; 27 subsequent DNI diagnoses, 2.0% overall and 4.7% of those with records), with these cases retained as index-negative and the rationale stated. (Sections 2.2 ¶2, 2.3 ¶3, 3.1 ¶3)
Statistical methods	12(e)	Describe any sensitivity analyses	CT-defined subgroup analysis (uniform anatomical reference), symptom-duration-adjusted models, ANC-versus-WBC model exchange, and marker-specific paired AUC comparisons in matched subsets (Supplementary Table

			S4). (Sections 2.6, 3.3–3.5)
Participants	13(a)	Report numbers of individuals at each stage of the study	Section 3.1, first paragraph, and Figure 1: 2,497 screened; exclusions (trauma 659, isolated foreign body 145, tumor-related 45, iatrogenic 8); final cohort 1,640. (p.6)
Participants	13(b)	Give reasons for non-participation at each stage	Figure 1 lists exclusion counts by reason. (p.6)
Participants	13(c)	Consider use of a flow diagram	Figure 1 shows four terminal outcome branches: CT-defined DNI (n=223), CT without confirmed DNI (n=439), confirmed DNI without CT (n=50), and no confirmed DNI without CT (n=928). (p.6)
Descriptive data	14(a)	Give characteristics of study participants and information on exposures and potential confounders	Table 1: age, sex, chief complaints, vital signs, laboratory values, consultation, admission, and drainage procedures by DNI status. (p.7)
Descriptive data	14(b)	Indicate number of participants with missing data for each variable of interest	Model-specific complete-case samples and event counts are reported, and included-versus-excluded patients are compared in Supplementary Table S3. (Sections 3.3–3.4; Tables 2–3 footnotes)
Descriptive data	14(c)	Summarize follow-up time	Seven-day institutional follow-up of index-negative patients (with a 30-day window examined as an additional internal check); ENT consultation rate in non-CT patients reported (151/978; 15.4%). (Sections 2.2–2.3, 3.1–3.2)
Outcome data	15	Report numbers of outcome events or summary measures	Confirmed DNI in 273/1,640 (16.6%); CT-defined DNI in 223/662 (33.7%); non-CT confirmed DNI in 50/978. (Section 3.1; Table 1)
Main results	16(a)	Give unadjusted estimates and, if applicable, confounder-adjusted estimates and their precision	Table 1 (unadjusted comparisons with p-values), Table 2 (adjusted ORs with 95% CIs), Table 3 (incremental ΔAUC with CI, likelihood-ratio tests, calibration), marker-specific IQR-scaled associations, and Supplementary Table S4 (paired AUC comparisons). (pp.7–9)
Main results	16(b)	Report category boundaries when continuous	Continuous variables were not categorized (original/log2 scales retained); marker-specific analyses are

		variables were categorized	reported per IQR with the absolute IQR value for each marker shown. (Section 2.6; Table 3 footnote)
Main results	16(c)	If relevant, consider translating estimates of relative risk into absolute risk for a meaningful time period	Absolute frequencies (e.g., 16.6%) and discrimination/calibration metrics (Brier score 0.170→0.144; calibration slope 0.823→0.908) are reported; the manuscript explicitly states that performance is insufficient for stand-alone use, preventing over-interpretation. (Sections 3.1, 3.4; Abstract)
Other analyses	17	Report other analyses done	Marker-specific analyses of derived indices (NLR, PLR, SII, SIRI, AISI, CAR, NPAR, CALLY, HALP) with paired AUC comparisons; abscess-versus-non-abscess phenotype analysis; CT-versus-non-CT comparisons (Supplementary Tables S1, S2, S4). (Sections 3.2, 3.5)
Key results	18	Summarize key results with reference to study objectives	Discussion, first and second paragraphs, and Conclusions (Section 5): incremental value of ANC and CRP beyond clinical characteristics, no advantage of derived indices, and reproduction in the CT-defined subgroup. (pp.9, 11)
Limitations	19	Discuss limitations of the study, taking into account sources of potential bias or imprecision	Discussion, ninth paragraph: retrospective single-center design; partial verification bias with expected direction; unavailability of outside-institution prescriptions and follow-up; interobserver agreement (kappa) not quantified; absence of external validation. (pp.9–10)
Interpretation	20	Give a cautious overall interpretation of results considering objectives, limitations, multiplicity of analyses, results from similar studies, and other relevant evidence	Discussion, eighth paragraph: cautious interpretation ("refine, rather than replace"), airway-stabilization priority, the indispensable anatomical role of CT, and explicit statements that no threshold, score, or imaging decision rule was derived. (pp.9–10)
Generalisability	21	Discuss the generalisability	Discussion, ninth paragraph: limited generalizability of a single tertiary-

		(external validity) of the study results	center, imaging-selected cohort; external validation identified as required future work. (p.10)
Funding	22	Give the source of funding and the role of the funders for the present study	Declarations: "Funding: This work was supported by INHA UNIVERSITY Research Grant." (p.11)
