# Supplementary material for: Laboratory Markers Associated with Deep Neck Infection in Emergency Department Patients with Throat or Neck Symptoms
Source: Diagnostics (Basel). 2026 Sep 17;16(18):3016. doi: 10.3390/diagnostics16183016 (PMC13605789; doi:10.3390/diagnostics16183016)
Supplement: Supplementary file 1 [file diagnostics-16-03016-s001.zip › diagnostics-4492460-supplementary tables.pdf]

Supplementary Table S1. Outcome ascertainment in the 50 confirmed DNI cases without study-institution CT.

Panel A. Confirmation pathway among confirmed DNI cases without study-institution CT (n=50).

Characteristic	n	%
Outside CT reportedly performed before transfer	35	70.0
In-house endoscopy/fibroscopy performed	50	100.0
Primary in-house confirmation: endoscopy/fibroscopy	44	88.0
Primary in-house confirmation: ultrasonography + aspiration/drainage	6	12.0
Additional procedural/operative corroboration	11	22.0

External CT images could not be accessed through the institutional system and were unavailable for study-specific image adjudication. External CT history was therefore treated as clinical context rather than study CT confirmation; the research classification relied on qualifying in-house evidence.

Panel B. ENT-documented anatomical regions among these 50 cases.

Anatomical region	n	%
Parapharyngeal	25	50.0
Retropharyngeal/posterior pharyngeal	16	32.0
Supraglottic/laryngeal	7	14.0
Submandibular	5	10.0
Sublingual/tongue base/floor of mouth	2	4.0
Other pharyngeal/velopharyngeal	2	4.0
Submental	1	2.0
Prevertebral	1	2.0

Anatomical regions were abstracted from the locations documented by the treating otolaryngology team during endoscopic, ultrasonographic, or procedural assessment. Categories were non-mutually exclusive and are descriptive rather than CT-equivalent fascial-plane adjudications.

Supplementary Table S2. Sensitivity analyses of the incremental value of ANC and CRP.

Analysis	N	Events	Clinical CV AUC	+ANC/CRP CV AUC	ΔAUC (95% CI)
Primary analysis	1,166	268	0.644	0.788	0.144 (0.109–0.179)
7-day delayed DNI classified positive	1,166	287	0.628	0.767	0.139 (0.104–0.172)
Broader outcome including isolated PTA/ITA	1,166	368	0.638	0.786	0.148 (0.116–0.180)
Inverse-probability-of-complete-case weighting	1,166	268	0.676	0.815	0.139 (0.106–0.174)

The first three analyses used the same complete-case predictor set with alternative outcome definitions. The weighted analysis used the observed complete cases with inverse-probability weights estimated from age, sex, fever, sore throat/odynophagia, neck pain, and CT-evaluation status. AUC confidence intervals for ΔAUC were obtained by paired bootstrap resampling. PTA/ITA, peritonsillar/intratonsillar abscess.

Supplementary Table S3.

Panel A. CT-evaluated versus patients without study-institution CT.

Variable	CT-evaluated (n=662)	No study-institution CT (n=978)	p value
**Demographic characteristics**			
Age, years	41.00 (30.00-56.00)	34.00 (26.00-53.00)	<0.001
Male sex	387 (58.5)	430 (44.0)	<0.001
**Medical history**			
Hypertension	90 (13.6)	132 (13.5)	0.999
Diabetes mellitus	61 (9.2)	85 (8.7)	0.747
Dyslipidemia	29 (4.4)	35 (3.6)	0.438
Malignancy	23 (3.5)	38 (3.9)	0.761
**Clinical presentation**			
Time from symptom onset to ED arrival, h	48.00 (17.47-94.16)	19.93 (1.14-71.43)	<0.001
Fever	32 (4.8)	56 (5.7)	0.432
Sore throat or odynophagia	477 (72.1)	765 (78.2)	0.004
Neck pain	183 (27.6)	210 (21.5)	0.004
Neck or submandibular swelling	11 (1.7)	5 (0.5)	0.020
Dyspnea or stridor	10 (1.5)	5 (0.5)	0.037
Voice change or hoarseness	2 (0.3)	2 (0.2)	0.666
**Initial vital signs**			
Temperature, degrees C	37.00 (36.60-37.70)	36.85 (36.50-37.30)	<0.001
Systolic blood pressure, mmHg	136.00 (121.00-151.00)	130.00 (119.00-147.00)	<0.001
Diastolic blood pressure, mmHg	82.00 (72.00-92.00)	80.00 (70.00-89.00)	0.035
Heart rate, /min	93.00 (82.00-105.00)	89.00 (79.00-101.00)	<0.001
Respiratory rate, /min	18.00 (18.00-20.00)	18.00 (18.00-18.00)	<0.001
Oxygen saturation, %	98.00 (97.00-99.00)	98.00 (97.00-99.00)	0.018
**Initial laboratory findings**			
WBC count, x10^9/L	11.20 (8.44-14.58)	8.71 (6.42-11.72)	<0.001
ANC, x10^9/L	8.58 (6.10-11.77)	6.04 (4.17-9.04)	<0.001
Platelet count, x10^9/L	226.00 (188.00-269.00)	228.00 (192.00-270.00)	0.286
ESR, mm/h	22.00 (9.00-37.00)	11.00 (5.00-26.00)	<0.001

CRP, mg/dL	4.78 (1.63-9.85)	1.32 (0.29-3.99)	<0.001
Albumin, g/dL	4.10 (3.90-4.40)	4.20 (4.00-4.40)	0.002
**Derived inflammatory indices**			
NLR	6.32 (3.67-10.28)	3.95 (2.27-7.14)	<0.001
PLR	166.36 (123.23-218.44)	151.03 (111.85-217.12)	<0.001
SII	1482.58 (831.55-2352.37)	889.60 (485.01-1589.42)	<0.001
SIRI	3.94 (1.86-7.08)	1.88 (0.78-4.32)	<0.001
AISI	882.21 (423.27-1590.87)	434.08 (183.35-977.02)	<0.001
CAR	1.15 (0.38-2.46)	0.31 (0.07-0.95)	<0.001
NPAR	18.72 (16.48-20.86)	17.08 (14.60-19.37)	<0.001
CALLY	1.16 (0.46-4.08)	3.51 (1.02-17.25)	<0.001
HALP	36.27 (25.21-50.18)	34.96 (23.47-52.44)	0.518
**Clinical course and management**			
Antibiotic prescription within previous 7 days	17 (2.6)	21 (2.1)	0.618
Corticosteroid prescription within previous 7 days	10 (1.5)	16 (1.6)	1.000
ENT consultation	535 (80.8)	151 (15.4)	<0.001
Hospital admission	297 (44.9)	117 (12.0)	<0.001
Confirmed drainage procedure	44 (6.6)	18 (1.8)	<0.001

Values are median (interquartile range) or n (%). The variables shown characterize clinical selection into study-institution CT imaging. Pretreatment variables reflect prescriptions recorded within the study institution during the 7 days preceding the index visit; outside prescriptions were unavailable.

Panel B. Anatomical distribution.

Anatomical category	n	% of CT-defined DNI
Parapharyngeal	103	46.2
Submandibular	62	27.8
Retropharyngeal	58	26.0
Preepiglottic	17	7.6
Paraglottic	14	6.3
Sublingual	6	2.7
Prevertebral	3	1.3
Parotid	2	0.9
Danger	2	0.9
Carotid	2	0.9
Masticator	1	0.4
Anterior visceral/pretracheal	1	0.4
Multiple-space involvement	59	26.5

Anatomical spaces were coded as non-mutually-exclusive categories.

Supplementary Table S4. Anatomical distribution and abscess versus non-abscess morphology of CT-defined DNI (n=223).

Panel A. Morphology classification

Morphology	n	%
Definite deep-space abscess	72	32.3
Non-abscess DNI	151	67.7

Panel B. Laboratory markers and age- and sex-adjusted associations with definite abscess.

Marker	Non-abscess DNI, median (IQR) (n=151)	Definite abscess, median (IQR) (n=72)	Unadjusted p value	Age/sex-adjusted OR (95% CI)	Adjusted p value
WBC, $\times 10^9$/L	12.27 (9.43–15.17)	14.05 (10.87–17.41)	0.004	1.52 (1.14–2.03)	0.004
ANC, $\times 10^9$/L	9.62 (7.35–12.52)	11.31 (8.71–15.05)	0.008	1.50 (1.12–2.03)	0.007
CRP, mg/dL	5.59 (3.04–11.30)	7.34 (2.62–14.07)	0.239	1.11 (0.94–1.32)	0.220
ESR, mm/h	26 (11–39)	27 (17.5–44.3)	0.166	1.23 (0.98–1.54)	0.070

Unadjusted comparisons used the Mann–Whitney U test. Adjusted odds ratios were estimated in separate logistic regression models containing age, sex, and the indicated marker; the outcome was definite abscess versus non-abscess DNI among CT-defined DNI cases. WBC and ANC were modeled on their original scales and reported per 5 $\times 10^9$/L increase, whereas CRP and ESR were log2-transformed and reported per two-fold increase. These phenotype analyses were exploratory.

Supplementary Table S5. Comparison of patients included in and excluded from the primary incremental analysis

Characteristic	Included complete cases (n=1,166)	Excluded because of ≥1 missing model variable (n=474)
Proportion of full cohort	71.1%	28.9%
Confirmed DNI	268 (23.0%)	5 (1.1%)
No confirmed DNI	898 (77.0%)	469 (98.9%)
CT-evaluated	623 (53.4%)	39 (8.2%)
Not CT-evaluated	543 (46.6%)	435 (91.8%)

Supplementary Table S6. Comparison of discriminatory performance of derived inflammatory indices with ANC and CRP

Index	N (events)	Index AUC	Versus ANC: ANC AUC; ΔAUC (95% CI)	Versus CRP: CRP AUC; ΔAUC (95% CI)
NLR	1176 (269)	0.691	0.716; Δ -0.025 (-0.050 to +0.000)	0.742; Δ -0.051 (-0.085 to -0.015)
PLR	1174 (269)	0.565	0.715; Δ -0.151 (-0.195 to -0.107)	0.743; Δ -0.178 (-0.222 to -0.135)
SII	1174 (269)	0.682	0.715; Δ -0.033 (-0.055 to -0.009)	0.743; Δ -0.061 (-0.098 to -0.025)
SIRI	1176 (269)	0.711	0.716; Δ -0.005 (-0.024 to +0.015)	0.742; Δ -0.031 (-0.062 to +0.001)
AISI	1174 (269)	0.701	0.715; Δ -0.014 (-0.035 to +0.004)	0.743; Δ -0.042 (-0.077 to -0.009)
CAR	1173 (269)	0.742	0.717; Δ +0.026 (-0.012 to +0.060)	0.743; Δ -0.001 (-0.003 to +0.002)
NPAR	1173 (269)	0.698	0.717; Δ -0.019 (-0.047 to +0.010)	0.743; Δ -0.045 (-0.078 to -0.012)

CALLY	1173 (269)	0.729	0.717; Δ +0.012 (-0.022 to +0.047)	0.743; Δ -0.014 (-0.025 to -0.004)
HALP	1171 (269)	0.570	0.716; Δ -0.146 (-0.191 to -0.099)	0.744; Δ -0.173 (-0.214 to -0.131)

All AUCs within each row were calculated in the same subset of patients with complete data for the derived index, ANC, and CRP; therefore, sample sizes differ from those in Table 4 and the conventional-marker models. CALLY and HALP were oriented so that lower values indicated higher DNI probability. Confidence intervals were obtained by paired bootstrap resampling. No composite index showed a consistent discriminatory advantage over both ANC and CRP.
